# Role and Relevance of Cerebrospinal Fluid Cells in Diagnostics and Research: State-of-the-Art and Underutilized Opportunities

**DOI:** 10.3390/diagnostics12010079

**Published:** 2021-12-30

**Authors:** Ferdinand Otto, Christine Harrer, Georg Pilz, Peter Wipfler, Andrea Harrer

**Affiliations:** 1 Department of Neurology, Paracelsus Medical University, Christian-Doppler-Klinik, 5020 Salzburg, Austria; f.otto@salk.at (F.O.); christine.harrer@stud.pmu.ac.at (C.H.); georg.pilz@salk.at (G.P.); p.wipfler@salk.at (P.W.); 2 Department of Dermatology and Allergology, Paracelsus Medical University, Landeskrankenhaus, 5020 Salzburg, Austria

**Keywords:** CSF immune cells, cytology, flow cytometry, immune phenotyping

## Abstract

Cerebrospinal fluid (CSF) has recently experienced a revival in diagnostics and research. However, little progress has been made regarding CSF cell analysis. For almost a century, CSF cell count and cytomorphological examination have been central diagnostic parameters, with CSF pleocytosis as a hallmark finding of neuroinflammation and cytology offering valuable clues regarding infectious, autoimmune, and malignant aetiologies. A great deal of information, however, remains unattended as modern immune phenotyping technologies have not yet been broadly incorporated into routine CSF analysis. This is a serious deficit considering the central role of CSF cells as effectors in central nervous system (CNS) immune defence and autoimmune CNS processes, and the diagnostic challenges posed by clinically overlapping infectious and immune-mediated CNS diseases. Here, we summarize historical, specimen-intrinsic, methodological, and technical issues determining the state-of-the-art diagnostics of CSF cells and outline future perspectives for this underutilized window into meningeal and CNS immunity.

## 1. Introduction

Cerebrospinal fluid (CSF) is a clear colourless liquid that surrounds the brain and spinal cord and plays a vital role in central nervous system (CNS) homeostasis [1]. As far back as the ancient Egyptians and Greeks, the existence of human CSF has been recognized; however, its physiological relevance was unclear. In the 1400s and 1500s, anatomists, such as DaVinci and Vesalius, were among the first to describe the structure of the brain and the ventricular system [2]. In the early 20th century, neurosurgeons Harvey Cushing (1914) and Walter Dandy (1919) identified the choroid plexus as the origin of CSF [3,4]. Today it is well established that CSF encompasses the entire CNS from the brain to the spinal cord, providing buoyancy and facilitating substance distribution to and from brain parenchyma for nourishment and waste disposal [5]. Ultrafiltrated by the choroid plexus from blood into the ventricular system, CSF enters the subarachnoid space by bulk-flow and diverges, flowing partly down the spine to the lumbar sac and partly over the surface of the brain. CSF exits the subarachnoid space at the arachnoid granulations into the blood and through the nasal cribriform plate or via spinal nerve roots into the lymphatic system [5].

Speaking about CSF usually refers to the fluid itself. However, cerebrospinal fluid also contains cells, namely a special selection of blood-derived lymphocytes and monocytes. These cells are capable of crossing the blood–CSF barrier (BCSFB) of the choroid plexus, which allows minimal entry of immune cells and macromolecules, such as antibodies, in steady-state [6]. Immune cells in the CSF are normally extremely few in numbers, predominated by central memory CD4+ T cells, and are supposedly committed to CNS immune surveillance. These cells patrol the subarachnoid space for pathogens and at least partly return to secondary lymphoid organs [7,8,9,10,11].

The subarachnoid space is part of the leptomeninges and contains immune sentinels and non-fenestrated blood vessels, which cover and traverse the brain surface, encased in pial sheaths with CSF flowing along in the perivascular spaces [12]. The functional relevance of the leptomeningeal barrier for CNS immune surveillance has only recently been recognized [13]. Thus, cerebrospinal fluid is a highly important immune interface between the meninges, CNS, and periphery. It steadily flows through the subarachnoid space in direct touch with resident dendritic cells and long-lived pial macrophages. Both sterile and infectious triggers can instigate meningeal inflammation, resulting in the degradation of the glia limitans followed by immune cell infiltration of the CNS parenchyma and a rise of neurologic disorders [12].

Due to its CNS-ensheathing proximity, accessibility, and obvious role in CNS immune surveillance, much attention is paid to CSF in laboratory diagnostics for neurological disorders—a development largely driven by German pathologists, clinicians, and researchers during the past century [14]. Viral infections are the most common causes of meningitis, encephalitis, and meningoencephalitis [15], and suspected infectious or inflammatory CNS diseases are leading indications for lumbar punctures [16]. Pleocytosis in CSF and intrathecal adaptive immune activities, in particular local antibody production, are pathological features commonly observed in neuroinflammation [17], and are assessed by CSF cell count, cytomorphological differential, and quantitative and qualitative measurements of CSF immunoglobulins since the second half of the 20th century [17,18,19].

While advances in laboratory techniques have broadened the indications and significance of CSF diagnostics by allowing investigating, for instance, an increasing repertoire of neuronal autoantibodies in suspected autoimmune encephalitis (AIE) and biomarkers of neurodegenerative diseases [20], little progress has been made regarding CSF cells. This is astonishing considering their central role in CNS immune surveillance as effectors and drivers of CNS immune defence as well as autoimmune CNS processes, in particular humoral adaptive immune activities, such as intrathecal antibody synthesis [21]. Furthermore, it is a serious deficit considering that infectious and immune-mediated CNS diseases can clinically overlap and instigate each other [22].

Why is this so? Here, in the quest for an answer, we outline the historical and state-of-the art methods of investigating CSF cells for diagnostic purposes and summarize technologically available yet underutilized opportunities for enhancing our understanding of meningeal CNS immunity.

## 2. The Challenge of Examining CSF Immune Cells—A History of Ups and Downs

Lumbar punctures were first performed in the 1890’s to reduce intracranial pressure in patients with hydrocephalus and tubercular meningitis [23,24]. At about the same time, the newly introduced Köhler illumination allowed for differentiation of cell-types by light field microscopy [25]. Thus, it quickly became clear that CSF contained few cells, which—dependent on the underlying pathology—differed in numbers and phenotype, and apparently were blood-derived leukocytes. However, the development of reliable and practicable methods for examining CSF cells turned out to be a big challenge, because centrifugation for cell enrichment, which was already a part of the protocol of the so-called French Method of 1901, damaged the sensitive CSF cells [26]. In 1904, Fuchs and Rosenthal developed their counting chamber method, which is still a state-of-the art method for quantifying CSF cells; however, it does not allow one to differentiate cells [27]. The following years were determined by trial and error, and involved CSF processing methods for cell preparation, such as cell sediment embedding in celloidin or ammonium sulphate precipitation of CSF, which all turned out to be too laborious and/or too damaging to the cells to be useful in clinical practice [28]. The breakthrough was half a century later, when Johannes Sayk developed a cell sedimentation chamber, which combined the principle of spontaneous sedimentation accelerated by a strip of absorbent paper [29]. This procedure was comparatively fast and cost-efficient, enabled CSF cell differentiation in high quality, and revolutionized CSF diagnostics in Europe. In the 1990’s, the Shandon cytocentrifuge, allowing a quick production of a cytological specimen with low cell loss, replaced the “Sayk chamber”, and is currently the standard method for CSF cytodiagnostics [28].

**Figure 1:**
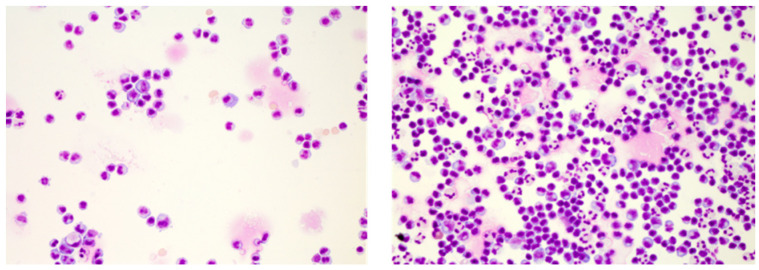
Cerebrospinal fluid (CSF cytology illustrating the impact of elapsed time on CSF cells. Shown are May–Grünwald stained CSF cells of an enterovirus meningitis case featuring an activated lymphocytic phenotype and 152 cells/µL after several hours of delayed processing (**left**) and a predominant lympho-monocytic activated phenotype with intermingled granulocytes and 299 cell/µL upon immediate processing (**right**). Cytology specimen kindly provided by the Department of Laboratory Medicine, Uniklinikum Salzburg, Paracelsus Medical University Salzburg (Chair: Univ-Prof Dr. Elisabeth Haschke-Becher).

With the methodological issues of cell preparation resolved, the time from bedside to the lab remained the most critical factor for investigating CSF cells. Ex vivo cerebrospinal fluid quickly turns toxic to its cells, thus severely limiting their lifespan [30]. Moreover, CSF cell subpopulations do not decay at an equal rate; granulocytes are affected first, followed by monocytes, and lastly lymphocytes. If not processed immediately, that is, within one to maximally two hours after lumbar puncture, this may severely alter the inflammatory phenotype of immune cells and result in misleading diagnostic cues. A mixed cell picture consisting of granulocytes, monocytes, lymphocytes, and activated forms indicating an infectious etiology, for example, may present as a lymphocytic pleocytosis with activated lymphocytes suggesting an autoimmune CNS process (Figure 1).

With the crucial role of timing in preanalytics established, reducing manual processing and speeding up analytics by introducing modern hematoanalysers capable of special fluidic acquisition modes was pursued. Although alluring and intensely investigated for CSF cells, automation reached no breakthrough in fully replacing the manual counting chamber method or cytospin preparations [31]. Again, CSF cells turned out to be too delicate a sample specimen, requiring special attention. Automation may be helpful for preliminary information about absolute cell numbers and whether granulocytes or mononuclear cells predominate; that is, in differentiating suspected bacterial from non-purulent meningitis. However, the precision of automated cell counts is only moderate at low CSF cell counts [32], and automated differentials either do not or unsatisfactorily detect cell pathologies, such as neoplastic cells, erythrophages, siderophages, and other reactive, activated, and slightly disintegrated cell morphologies [33]. Thus, examining CSF cells remains a manual relict requiring skilled hands and personnel.

## 3. Conventional CSF Cell Diagnostics

**Figure 2:**
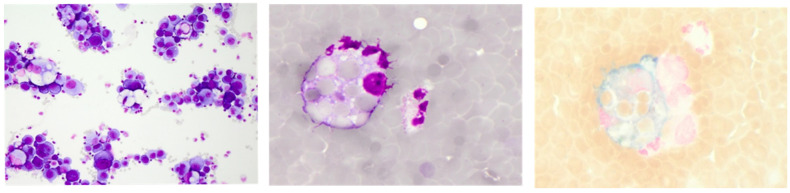
Representative CSF cytology illustrating neoplastic leptomeningeal spread with carcinoma cells (**left**, May–Grünwald staining) and an acute subarachnoid haemorrhage depicting an erythrophage (**middle**, May–Grünwald staining) and an erythrosiderophage (**right**, Berliner blue staining). Cytology specimen kindly provided by the Department of Laboratory Medicine, Uniklinikum Salzburg, Paracelsus Medical University Salzburg (Chair: Univ-Prof Dr. Elisabeth Haschke-Becher).

This brings us to the central role and relevance of CSF cell count and May–Grünwald stained cytology in the CSF diagnostic work-up of neurological disorders. Absolute CSF cell numbers frequently earn the most attention from clinicians, as they are available faster than cytology. In addition, if elevated, they are highly informative via the degree of elevation. However, only combined interpretation with cytomorphology allows accurate diagnostic clues about the underlying pathology [34]. For instance, in the case of suspected leptomeningeal neoplastic spread or subarachnoid haemorrhage (SAH), the detection of neoplastic cells or, respectively, erythro- and/or siderophages on cytoslides confirms the diagnosis irrespective of a normal or elevated CSF cell count (Figure 2).

**Figure 3:**
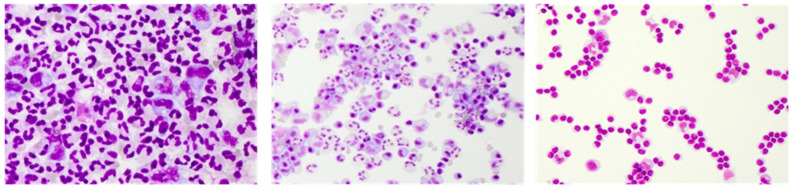
Representative May–Grünwald stained CSF cytology images of bacterial meningitis (**left**), viral meningitis (**middle**), and an autoimmune central nervous system (CNS) disease (**right**). Cytology specimen kindly provided by the Department of Laboratory Medicine, Uniklinikum Salzburg, Paracelsus Medical University Salzburg (Chair: Univ-Prof Dr. Elisabeth Haschke-Becher).

Normal CSF cell counts are below 5 cells/µL, consisting of mononuclear white blood cells with a majority of lymphocytes and fewer monocytes [33]. Cell count elevations are termed CSF pleocytosis and are a hallmark finding of neuroinflammation in suspected meningitis, autoimmune CNS disorder, or neoplastic leptomeningeal spread. Cerebrospinal fluid pleocytosis may be reactive, i.e., non-inflammatory by nature, occurring in conditions such as stroke, epileptic seizures, trauma, neurosurgical intervention, or acute SAH; however, it can also be the result of artificial blood contamination during the lumbar puncture procedure. Reactive pleocytosis usually presents with mild to moderate cell count elevations consisting of non-activated mononuclear cells and a more or less pronounced fraction of neutrophil granulocytes [35]. Massive granulocyte predominance along with very high CSF cell counts (>1000 cells/µL) are characteristic findings of bacterial meningitis (Figure 3) [17]. Viral meningitis can also present with high CSF pleocytosis (up to 1000 cells/µL); however, it primarily shows a mononuclear cell pattern, including activated lympho- and monocytes, and sometimes plasma cells (Figure 3) [17]. Similar cell counts also occur in aseptic bacterial infection caused, for instance, by *Mycobacterium tuberculosis, Listeria monocytogenes*, *Treponema Pallidum*, or *Borrelia burgdorferi*. In these cases, the cell picture is typically more varied, featuring numerous plasma cells, activated lympho- and monocytes and, more or less frequently, granulocytes [17,36]. Plasma cells, representing end-differentiated B cell lineage cells and professional antibody producers, are of particular interest in the differential diagnosis of autoimmune CNS disorders. Combined with mild to moderately elevated or even normal CSF cell counts, their occurrence in a clearly lymphocyte-dominated cell environment strongly supports the diagnosis of suspected autoimmune processes, such as multiple sclerosis (MS) or autoimmune encephalitis (AIE) (Figure 3) [17]. In suspected neurodegenerative disease, again, anything other than a normal finding regarding CSF cell count and cytology emphasizes the consideration a differential diagnosis [17].

**Figure 4:**
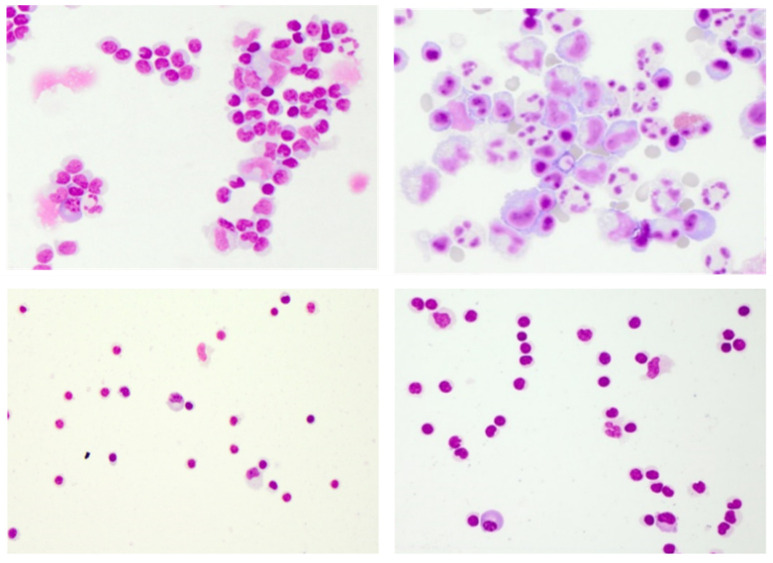
Limitations of conventional CSF cytology. May–Grünwald staining illustrating a case of Lyme neuroborreliosis with antibiotic pre-treatment resembling viral meningitis (**upper left**), an atypical case of Varicella Zoster Virus-meningitis with large hyperreactive cells and plasma cells resembling aseptic bacterial meningitis or Epstein-Barr Virus infection, possibly in malignant transformation (**upper right**), and the almost identical cytomorphological findings of the two autoimmune CNS diseases multiple sclerosis (**lower left**) and anti-N-methyl-D-aspartate receptor encephalitis (**lower right**). Cytology specimen kindly provided by the Department of Laboratory Medicine, Uniklinikum Salzburg, Paracelsus Medical University Salzburg (Chair: Univ-Prof Dr. Elisabeth Haschke-Becher).

Thus, the informative value of examining CSF cells is evident. However, dependent on the individual patient’s immune system, age, comorbidities, disease stage, and (pre-)medication, cytological findings may be less characteristic or even overlap between aetiologies. Aside from bacterial infection, granulocytes may also occur in the early stages of viral infections [17]. A lymphocytic predominance with intermingled plasma cells typically seen in autoimmune-related conditions may as well occur in the later stages of viral meningitis [37]. Antibiotic pre-treatment may change a highly activated lympho-monocytic picture with multiple plasma cells of a Lyme neuroborreliosis (LNB) towards a moderately activated appearance rather suggestive of viral infection (Figure 4). Both viral and aseptic bacterial infections can feature an accumulation of hyper-reactive plasma cells resembling malignant lymphoma [35].

The main limitation of conventional CSF cytology relates to it being confined to cell morphology, enabling no further differentiation of lymphocytes and monocytes than their activation state, and the occurrence of plasma cells as evidence of intrathecal B cell activity. Fortunately, CSF diagnostics is not built on CSF cytology alone; it also combines and integrates CSF cell count, protein analysis, antibody tests and indices, and microbiological and genome analysis.

Nevertheless, the clinical need for a broader understanding of the inflammatory CSF cell phenotype is underlined by an estimated 10–20% of unresolved meningoencephalitis cases that are believed to be non-infectious and immune-mediated, relating to AIE [38,39]. Knowledge about the exact identity and composition of effector cells holds great promise not only for improving the differentiation between infectious and autoimmune or within different autoimmune inflammatory CNS processes (Figure 4), but also for allowing for conclusions about disease stage and disease course.

## 4. Clinical Relevance of Immune Phenotyping of CSF Cells by Flow Cytometry

Flow cytometry was quickly recognized as a highly promising method to close the knowledge gap regarding the inflammatory immune cell phenotype. Complicating factors, such as the need for immediate processing combined with a limited availability of cells, did not dampen the rising enthusiasm for CSF immune cell phenotyping in the 1990’s, when early flow cytometers allowed 2–4 colour experiments.

**Figure 5:**
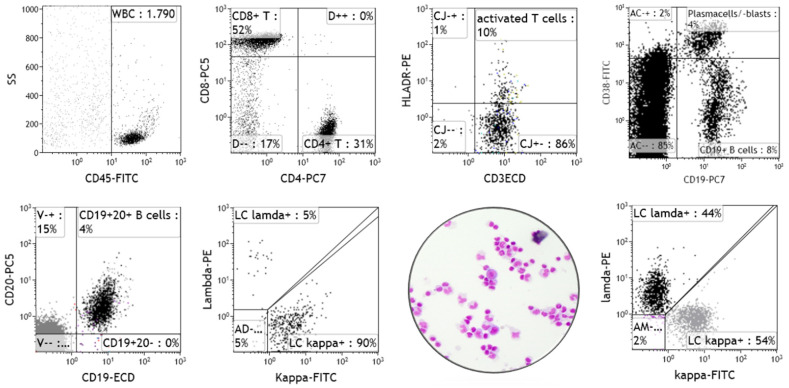
Flow cytometry of CSF cells (own data). Upper panel, from left to right: Shown are representative flow cytometry plots illustrating CD45+ CSF immune cells (WBC gate), mainly lymphocytes (SSC^low^, black population), the CD4+ and CD8+ T cell composition for calculating the CD4+/CD8+ T cell ratio, activated CD3+ T cells (upregulation of HLA-DR), and at the far right, CD19+ B cell activation in the form of plasma blasts (CD19^intermediate^CD38^bright^). The lower panel illustrates B cell light chain analysis results with the second plot showing kappa light chain clonality (ratio = 18) confirming suspected CNS B cell malignancy versus a normal kappa/lambda light chain distribution (ratio ~1) shown in the plot far right, and an un-ascertainable CSF cytology of suspected B cell lymphoma in between.

Those years produced pivotal studies that provided the basis of current knowledge about normal and inflammatory CSF immune cell phenotypes. One of the most intriguing findings was that CSF lymphocytes were not a random sample of blood-derived lymphocytes but were a special selection capable of migrating across brain barriers and proposedly involved in immune surveillance [40,41]. Compared to the blood, there was a selective enrichment of CD4+ T cells, fewer CD8+ T cells, and a lack of or very few B cells. [40,41,42,43]. Another highly relevant observation at that time was that alterations of the CD4/CD8 T cell ratio appeared to be diagnostically exploitable, as shifts towards CD8+ T cells supported the diagnosis of viral meningitis, whereas a shift towards CD4+ T cells supported the diagnosis of suspected MS [43]. Ultimately, a basic phenotyping for diagnostic purposes, analogous to peripheral blood, was introduced for CSF, including preliminary normal ranges of total and activated CD3+ T cells, CD4+ and CD8+ T cell subpopulations, B cells, NK cells, and the CD4/CD8 T cell ratio (Figure 5, upper panel) [40].

However, these early achievements suffered severely from a delay in technical advances for flow cytometry. Too many years with too few colour experiments for too few CSF cells (due to specimen-intrinsic cellular paucity) hampered both the broader translation into clinical diagnostics and further research. The situation was aggravated as lumbar punctures were considered invasive, and technological advances in brain imaging substituted some of the indications for CSF [16,44]. The overall effect was that CSF immune cell phenotyping was to some extent abandoned at a time, when flow cytometry technology and methodology started to boom.

Today, flow cytometry has evolved into an indispensable tool for disease and treatment monitoring of blood immune phenotypic markers in both clinical diagnostics and research [45]. New fluorescent dyes, high-throughput monoclonal antibody engineering technologies, the availability of a broad spectrum of fluorescent-labelled detection antibodies, and the development of multi-laser flow cytometers, with up to 30-color research applications and 10-color in vitro diagnostics (IVD) instruments, made this possible.

These advances, however, have only partially reached CSF diagnostics. In addition to specimen-intrinsic factors relating to the instability and paucity of CSF cells, clinical implementation of flow cytometry for CSF cells has suffered from: (i) low CSF collection volumes (lumbar punctures were considered invasive), (ii) lack of consensus protocols for preanalytics, processing, antibody panels, and gating strategies, and (iii) outsourcing of CSF diagnostics from specialized neuroimmunological to large central laboratories observable at least in German speaking European countries.

Today, the only widely accepted indication for diagnostic flow cytometry and its sole mention in the current S1 guidelines for CSF diagnostics of the German Society [14,17] are suspected haematological CNS malignancies for differentiating lymphocyte pleocytosis of an unknown cause from a meningeosis lymphomatosa [17]. In non-Hodgkin lymphoma (NHL), the estimated overall risk of CNS involvement is 4.2% [46]. Though relatively uncommon compared to the up to 30–40% CNS relapse detected in acute lymphoblastic leukemia [47], the prognostic and therapeutic significance is grave [48]. The risk of CNS relapse in NHL strongly depends on the type amounting up to 24.4% in high-grade NHL, lymphoblastic, and Burkitt’s NHL compared to 2–8% in low-grade NHL [46].

Conventional cytology has a rate of up to 60% false negative results [30]. Provided with a minimum of 5–10 mL CSF collection volume, multiparametric flow cytometry increases the detection rate of malignant lymphocytes with a high specificity and greater sensitivity to up to 86%. Therefore, combining the two methods is clearly recommended [30]. Investigating B cell monoclonality is of particular relevance, because B cell-NHL is the most frequent haematological malignancy affecting the CNS (Figure 5, lower panel) [17,30]. Relevant markers of immature or aberrant phenotypes include co-expression analysis of CD34, CD10, CD30, TdT, or CD5 in B-NHL, and CD34, CD10, CD30, TdT, or CD1a in suspected CNS involvement of peripheral T cell-NHL. The latter are a heterogenous group of mature T cell neoplasms and account for approximately 10–15% of NHL, with limited data regarding the incidence and risk factors for CNS involvement [49,50]. Except for adult T cell lymphoma/leukemia, the majority of peripheral T-NHL types have low malignant potential but can still cause meningeosis with pronounced alterations of the CD4/CD8 T cell ratio, high proportions of CD4+CD8+ double positive T cells, and the loss of expression of normally expressed markers, such as CD7 or CD5 [17].

The situation is even more difficult in primary CNS lymphoma (PCNSL), a highly aggressive form of B-NHL of the diffuse large B cell-type with manifestations exclusively in the CNS and not the periphery [51]. The likelihood of detecting meningeal spread at the first lumbar puncture is only 30%. Lymphoma cells in CSF are usually in low abundance, and the evidence of monoclonality alone is insufficient for diagnosis unless high clinically documented suspicion of PCNSL exists [14,52].

Investigating the CD4/CD8 T cell ratio, however, finds no mention in the current S1 CSF diagnostic guidelines. This may be because earlier findings highlighting the CD4/CD8 T cell ratio, i.e., if decreased, supportive of suspected viral infection and, if increased, supportive of suspected MS, were not confirmed by more recent studies [37,53]. In contrast, the CD4/CD8 T cell ratio turned out to be relatively constant across several neurological diseases (range 1.8–8.0), which included but were not restricted to: bacterial and viral meningitis, LNB, MS, headache, and idiopathic intracranial hypertension [37]. The only exceptions with significant decreased CD4/CD8 T cell ratios (<1.8) were observed in human immunodeficiency virus (HIV) patients and other conditions of immunosuppression including immunomodulatory drugs, such as natalizumab [37,54]. 

This does not mean that earlier studies reporting decreased CD4/CD8 T cell ratios associated to viral infection were inaccurate. Rather, it indicates that similar to CSF cytology, immune phenotyping also depends on disease stage and therapy. Thus, expansions of cytotoxic CD8 T cells occurring earlier in the infection may be limited to the initial phase, after which activated CD4+ T-helper cells, B cells, and plasma cells build up the pathogen-specific humoral immune response. This interpretation is supported by the presence of elevated B cell frequencies detected by flow cytometry [37], activated lymphocytes and plasma cells observed in CSF cytology, and the diagnostic relevance of pathogen-specific antibody tests in viral meningitis [14,17].

Nevertheless, the strikingly low CD4/CD8 T cell ratio of HIV patients (0.5–1.8) and the fact that it is difficult to clinically differentiate HIV encephalitis from opportunistic infections [37,55] is just one example that shows CSF immune phenotyping has clinical potential beyond detecting haematological CNS involvement. Another example is CD8+ encephalitis, an increasingly recognized condition that causes neurological and neurocognitive disorders in HIV positive patients [56]. Possible triggers involve infection, immune reconstitution inflammatory syndrome, and intrathecal HIV viral escape, as a result of suboptimal anti-retroviral treatment (adherence) or viral mutation [57]. Evidence of high numbers of CD8+ T cells and a pronouncedly reversed CD4/CD8 T cell ratio in CSF is highly informative, and high-dose corticosteroid treatment is essential for a favourable outcome [57].

In neurosarcoidosis, an increased CD4/CD8 T cell ratio in CSF (>5.0) has been intensely investigated as a surrogate marker, aiding in differential diagnosis for many years with inconclusive results. Of clinical relevance, however, may be recent findings reporting (i) a negative predictive value of 88%, if a CD4/CD8 T cell ratio <5.0 is combined with an absence of pleocytosis [58], (ii) that IL-6 elevations of >50 pg/mL combined with an increased CD4/CD8 T cell ratio indicate relapse in active neurosarcoidosis [59], and (iii) that activated CD4 T cells in CSF combined with plasma cells in blood differentiate neurosarcoidosis from MS [60].

Following the idea that immune phenotyping is helpful for differential diagnoses in neuroinflammation, also the analysis of other lymphocyte subsets, e.g., B cells and NK cells, could be of help. Patients with HIV, LNB, bacterial meningitis, and MS showed differences in elevated fractions of B cells, with the highest elevations seen in LNB, and patients with viral meningitis showed significantly higher NK cell frequencies than MS patients [37].

To summarize, immune phenotyping of CSF cells for indications other than haematological CNS malignancies appears to be helpful. However, it is mainly restricted to neurological centres equipped with on-site neuroimmunological laboratories, multicolour flow cytometers, and associated research groups, discussed in more detail further below in the text [7,30,54,60,61,62,63,64].

## 5. CSF Cells in Research: Revisited

Cerebrospinal fluid is currently experiencing a renaissance as a window into our CNS. This renewed interest was ignited by the rise of molecular techniques allowing valuable mechanistic insights into disease pathologies for diagnostic, therapeutic, and research purposes. The result was an increasing repertoire of neurodegenerative CSF biomarkers and neuronal antibodies, the detection of previously unknown pathogens, and the opportunity of analysing CSF as liquid biopsy in suspected CNS malignancies, which altogether broadened the indications for lumbar punctures. The concurrent development and application of atraumatic needles reducing complications on the one hand, and the guideline recommendations for improved atraumatic lumbar punctures on the other hand, helped to expand acceptance that lumbar punctures in general are safe procedures [17,20]. This again peaked in studies that showed CSF collection volumes up to 30 mL are well tolerated, which are the basis of current guideline recommendations to routinely collect at least 10 mL for comprehensive high quality CSF diagnostics, and additional consensus guidelines for high-quality CSF processing for biomarker research [14,17,65]. Further opportunities arose by commercial availability of special CSF cell stabilization tubes, such as the TransfixTM/EDTA sample storage tubes (Cytomark, Buckingham, UK), which promise to stabilize cell surface antigens and prevent cellular degradation of major CSF cell subsets for up to 14 days. To our knowledge, CSF cell preservation is mainly used for detecting CNS infiltration of haematologic malignancies [48,66], and applicability for any other specific surface marker of interest requires prior testing.

In short, the combination of technological advances broadening indications for lumbar punctures and the higher CSF collection volumes alleviating limitations regarding the availability of sufficient CSF brought unforeseen opportunities for investigating CSF cells. Single cell high-throughput technologies allowing cell-type specific proteomic, transcriptomic, and genomic analyses appear as particularly promising methods to advance our understanding of intrathecal inflammatory CNS processes and, ultimately, CSF cytodiagnostics [67]. These single cell technologies include multi-dimensional flow cytometry and cell sorting, mass cytometry, single cell sequencing, and sequencing the receptor repertoire of B and T cells. Lanz et al. have comprehensively reviewed their potential and limitations for investigating CSF cells [67].

Multiparametric flow cytometry, for instance, is broadly accessible and widely used; however, it is limited by spectral overlap of fluorescent-labelled detection antibodies. Hence, it is very useful for phenotyping CSF cell heterogeneity by width rather than depth. Mass cytometry, in contrast, appears perfect for high dimensional, deep immune profiling into CSF cell subset diversity because the use of rare metals for detection obviates the spectral overlap of fluorophores [68]. This technology recently earned attention in CSF cell research, with the first publications indicating its feasibility. This is quite exciting considering the fact that the majority of available protocols demand huge cell numbers and freezing of cells [69,70], both major limitation regarding CSF cells. The latter though lately is being intensely investigated for its applicability on sensitive CSF cells (bioRxiv doi.org/10.1101/2021.09.15.460354). 

Unforeseen opportunities provide the rapid emergence of single-cell sequencing technologies allowing the production of high-throughput transcriptomic and, latest, surface expression data, which enables the identification of specific immune cell subsets and cell-type specific regulation patterns. First studies already successfully employed single-cell RNA sequencing (scRNAseq) for investigating CSF cells. One reported the compartment-restriction of T follicular helper cells in the CSF of MS patients, and another the occurrence of neurodegenerative disease-associated microglia-like cells in the CSF of patients with HIV [71,72].

Next-generation sequencing (NGS) is the method of choice when it comes to investigating the B cell receptor (BCR) and T cell receptor (TCR) repertoires. In fact, the intrathecal BCR repertoire has been intensely investigated in CSF and compared to blood, lymph nodes, and meningeal B cell accumulations in patients with MS. These results provided the basis of the concept that class-switched memory B cells and plasmablasts traffic across the blood–brain barrier [73,74,75]. Notably, NGS-based lineage analysis of CSF B cell repertoires pre and post natalizumab and fingolimod treatment of MS patients revealed pronounced differences between the two MS drugs on the intrathecal clonal expansion of B cells [76]. Besides MS, similar questions regarding BCR and TCR repertoire sequencing certainly apply to and are investigated in other neuroimmunological diseases, such as AIE [67].

The targeting of meningeal and CNS immunity with single cell technologies is developing at high speed. As an alternative to NGS, an approach combining scRNA-TCRseq of CSF cells with unbiased bioinformatics approach was recently successfully applied to quantify and visualize clonally expanded CSF T cells of patients with neurodegenerative diseases to pin down the intrathecal adaptive immunity in neurodegeneration [77].

It is obvious that these technologies hold great promise for a deeper understanding of intrathecal cellular processes and future cytodiagnostics. However, substantial further efforts and developments towards standardized protocols, multiplexing, and barcoding technologies allowing the pooling of samples from different individuals are necessary in order to gain high quality results despite the limitations of CSF cell paucity. Another important step is the inclusion of CSF investigation in clinical trials. This has been increasingly postulated as a necessity for new knowledge formation that is condensable into diagnostically exploitable formats [67,78,79].

## 6. Perspectives for Translation into CSF Diagnostics

From all the above mentioned single-cell technologies, immune phenotyping by flow cytometry currently has the greatest potential for advancing CSF cytology by translating new findings into clinical diagnostics. Flow cytometers are widely available and the costs are comparably low. Standardized protocols exist for clinical and trial settings, and shared platform activities help with optimized panel design and panel recommendations to improve data quality and reproducibility in research. Moreover, flow cytometry allows for the analysis of low cell numbers and can reliably identify T-lymphocytes even if the cell count is lower than 5 leukocytes/µL [30,67,80,81].

Accordingly, a great deal of effort has been dedicated to the special considerations and needs of CSF flow cytometry. De Graaf et al. [30], for instance, outlined the technical pitfalls related to low cellularity, the rapid decline of cells, unspecific binding of fluorochromes, and about how few events may be considered as a representative population of a cell subset. The authors also proposed a means of stabilizing CSF cells by adding serum-containing media [82]. Han et al. [63] illustrated the differences in the forward scatter and side scatter properties of CSF immune cells compared to their counterparts in peripheral blood, which is highly relevant knowledge for the correct gating of CSF cells.

Furthermore, a great deal of effort has been dedicated to identifying cellular biomarkers of neurological diseases and disease activity. Alvermann et al. [78] comprehensively reviewed the published evidence about cellular alterations, migration, and activation markers of CSF immune cells in MS, where a plethora of evidence revealed the heterogeneity of MS pathogenesis. More or less all CSF immune cell subpopulations were affected, including Th1 and Th17 subsets, regulatory T cells, T cell activation and B cell differentiation states, NK cells, monocytes, and dendritic cells [78].

CSF immune phenotyping data for MS patients is quite extensive. Though comparably modest in numbers, the knowledge gain obtainable through flow cytometry of CSF cells is increasingly recognized and applied in order to investigate other neurological diseases. These diseases include paraneoplastic disorders [30,79,83], cerebral vasculitis [84], non-paraneoplastic cerebellar ataxia [85], paediatric onset neuromyelitis optica [86] and anti-NMDAR-encephalitis [87], neurosarcoidosis [60], stroke [88], dementia [89], temporal lobe epilepsy [90], Susac syndrome [62], inflammatory neuropathies [60], tick-borne encephalitis [91], and HTLV-1-associated myelopathy/tropical spastic paraparesis [61], amongst others.

Increasing efforts further aim at deciphering the intrathecal inflammatory milieu by integrating cellular and humoral parameters, such as cytokines and chemokines [53,61,64,92]. Intrathecal immune networks involving B cells are of particular immune pathogenic relevance. Their presence and activity in the form of intrathecal antibody synthesis are a common theme and hallmark of infectious and immune-mediated inflammatory CNS disorders that has been diagnostically exploited for almost a century, while being poorly understood.

Calculating immune cell ratios was identified early as a useful tool for capturing additional information from immune phenotyping data. A shift of the CD4/CD8 T cell ratio reportedly may help to differentiate between possible CD4+ T cell dysregulation in CNS autoimmunity from a cytotoxic CD8+ T cell anti-viral immune defence [40,43] and an increased B cell/monocyte ratio hinting to relapsing–remitting MS [63]. Furthermore, an increase of the T cell/monocyte ratio helps to identify patients at risk of converting from a clinically isolated syndrome to definite MS [93].

Today, more sophisticated algebra in the form of modern computational methods promise profound advances via integrated data analysis of routine CSF diagnostic and multiparameter CSF immune phenotyping data. One of the first reports by Han et al. [63] investigated the relationship between immune phenotyping data and clinical diagnosis by unsupervised clustering. The results were clusters characterized by immune cell numbers and the dominance of either an innate or adaptive immune phenotype, which fitted well with the neurological diagnoses [63]. More recently, Heming et al. [92] applied a novel dimension reduction technique to CSF flow cytometry data from patients with inflammatory neuropathies and identified intra-disease heterogeneity suggestive of distinct disease mechanisms in the subgroups. Thus, algorithm-based methods appear to be highly promising in capturing the biology of the immune processes as a basis for targeted panel design and translation into clinical practice.

## 7. Final Comments

To summarize, CSF cells represent an important window into CNS disorders. These cells are intensely investigated but still underutilized in clinical CSF diagnostics. To date, not a single immune phenotyping biomarker, other than suspected lymphomatous malignancy, has been included in the consensus guidelines for aiding in differential diagnosis, individual prognosis, or treatment decisions. Besides the restricted time-points of lumbar punctures in the diagnostic work-up, major hurdles include specimen-intrinsic characteristics, such as low cell count and quick decay. This complicates the validation of findings in controlled settings of multicentre studies and the inclusion of immune phenotyping of CSF cells in clinical trials. In contrast, emerging efforts towards the qualitative cryopreservation of CSF, which, combined with modern molecular and immunologic technologies and computational methods, will accelerate the speed towards unforeseen insights into meningeal and CNS immune processes and further translation into clinical routine. With immune phenotyping by flow cytometry, a routine-qualified diagnostic method at hand, the revival of CSF cell analysis in clinical practice is highly feasible.

## Data Availability

Not applicable.
